# Nightlife clusters of coronavirus disease in Tokyo between March and April 2020

**DOI:** 10.1017/S0950268820002496

**Published:** 2020-10-13

**Authors:** S. Takaya, S. Tsuzuki, K. Hayakawa, A. Kawashima, A. Okuhama, K. Kanda, T. Suzuki, Y. Akiyama, Y. Miyazato, S. Ide, K. Nakamura, H. Nomoto, T. Nakamoto, S. Hikida, J. Tanuma, K. Ohara, T. Ito, T. Baba, K. Yamamoto, M. Ujiie, S. Saito, S. Morioka, M. Ishikane, N. Kinoshita, S. Kutsuna, N. Ohmagari

**Affiliations:** 1 Disease Control and Prevention Center, National Center for Global Health and Medicine, Tokyo, Japan; 2 Faculty of Infectious and Tropical Diseases, London School of Hygiene and Tropical Medicine, London, UK; 3 School of Tropical Medicine and Global Health, Nagasaki University, Nagasaki, Japan; 4 AMR Clinical Reference Center, National Center for Global Health and Medicine, Tokyo, Japan; 5 Faculty of Medicine and Health Sciences, University of Antwerp, Antwerp, Belgium; 6 Infectious Disease Emergency Specialist Training Program, Ministry of Health, Labour, and Welfare, Tokyo, Japan; 7 AIDS Clinical Center, National Center for Global Health and Medicine, Tokyo, Japan; 8 Bureau of International Health Cooperation, National Center for Global Health and Medicine, Tokyo, Japan

**Keywords:** COVID-19, emerging infections, infectious disease epidemiology

## Abstract

We analysed associations between exposure to nightlife businesses and severe acute respiratory syndrome coronavirus 2 PCR test results at a tertiary hospital in Tokyo between March and April 2020. A nightlife group was defined as those who had worked at or visited the businesses. We included 1517 individuals; 196 (12.9%) were categorised as the nightlife group. After propensity score matching, the proportion of positive PCR tests in the nightlife group was significantly higher than that in the non-nightlife group (nightlife, 63.8%; non-nightlife, 23.0%; *P* < 0.001). An inclusive approach to mitigate risks related to the businesses needs to be identified.

Japan reported its first coronavirus disease (COVID-19) case in mid-January 2020; as of 25 August, 62 507 cases and 1181 deaths have been reported in a population of 127 million [1], of which 19 428 cases and 352 deaths occurred in Tokyo [2]. The country adopted a cluster-focused approach to tackle the COVID-19 outbreak and set up an expert team called the COVID-19 cluster response taskforce to analyse data gathered from local governments [3]. The unique focus on clusters was based on the finding that a small number of COVID-19 patients were responsible for multiple cases, thus forming patient clusters, in ‘three Cs’ (close contact in a closed and crowded space) environments [3]. The Japanese health authorities have emphasised the cluster concept and requested the public to avoid such ‘three Cs’ environments. Since mid-March, however, the number of cases without an epidemiological link had increased in urban areas [4]. On 30 March, the governor of Tokyo asked the citizens to refrain from visiting nightlife businesses based on an analysis by the taskforce that approximately 30% of the sporadic cases notified in Tokyo in the previous two weeks were suspected to be connected to these businesses [4, 5].

The National Center for Global Health and Medicine (NCGM) is a referral hospital for infectious diseases in Tokyo. The centre is 2 km away from Kabukicho, the biggest nightlife district in the country. Given the characteristics of the patients that the NCGM serves, those who underwent severe acute respiratory syndrome-coronavirus-2 (SARS-CoV-2) PCR testing at the hospital could reflect those involved in nightlife clusters. Therefore, the aims of the study are to describe the demography of nightlife clusters and analyse the association between exposure to nightlife businesses and SARS-CoV-2 PCR test results.

All patients who underwent a SARS-CoV-2 PCR test at the NCGM's outpatient clinic from 9 March to 26 April 2020 were included in the study. During the study period, the national testing criteria for SARS-CoV-2 PCR testing were those who had common cold symptoms or a fever ⩾37.5 °C for four days or more (two days or more for older individuals and those with underlying medical conditions), malaise or dyspnoea [6]. Nasopharyngeal samples were collected, and reverse transcription PCR tests were performed unless it was clearly indicated otherwise. If an individual had undergone more than one PCR test, the tests were counted individually. The following information about the included individuals was collected; age, sex, nationality, comorbidities (respiratory disease, cardiovascular disease, chronic kidney disease, chronic liver disease, diabetes mellitus, malignancy, autoimmune diseases and HIV infection), consultation date, symptoms onset, severity, known exposure to confirmed cases, overseas travel within one month of symptom onset, involvement as a healthcare worker (HCW) (medical or nursing care) and SARS-CoV-2 PCR test result. Severity was categorised as either asymptomatic, mild or severe based on the patients' condition on the consultation day; severe disease was defined as a case that needed hospitalisation on the same day. The nightlife group was defined as those who had worked for nightlife businesses until at least one month before symptom onset or had visited those businesses within one month of symptom onset. Nightlife businesses included bars and pubs, host and hostess clubs, nightclubs and live music clubs, karaoke parlours and commercial sex businesses, as per the taskforce's report [4].

The nightlife and non-nightlife groups were initially compared using Fisher's exact test, Kruskal−Wallis test or Mann−Whitney *U* test. The covariates were chosen based on clinical relevance. Comorbidity was considered as any of the eight diseases listed earlier. A multivariable logistic regression model was then developed to estimate a propensity score for the nightlife group. Age, sex, nationality, comorbidity, severity, day of illness, exposure, overseas travel and HCW were included in the model. Propensity score matching was performed using the nearest-neighbour matching with a caliper width of 0.25 [7]. The standardised difference was used to measure covariate balance, and an absolute standardised difference above 10% was interpreted as a meaningful imbalance.

All statistical analyses were performed using Stata 15 (Stata Corp., College Station, TX, USA) and R, version 3.6.2. A two-tailed *P* < 0.05 was considered statistically significant. This study was approved by the NCGM ethics review board (NCGM-G-003537-00) and conducted in accordance with the approved guideline. Informed consent was obtained in the form of opt-out and the ethics review board approved this form of consent.

During the study period, 1517 PCR tests were performed for 1489 individuals at the NCGM. The number of PCR tests performed at the NCGM accounted for 15.7% (1517/9679) of the number of tests performed for diagnostic purposes in Tokyo during the same period [2]. The overall proportion of positive tests at the NCGM was not different from that in Tokyo (NCGM 21.9% [332/1517]; Tokyo 21.0% [2035/9679]; *P* = 0.372). The changes in the weekly proportions of positive tests over seven weeks were also not different by Spearman's correlation test (*ρ* = 0.96, *P* = 0.003).

Out of 1517 individuals, 196 (12.9%) were included in the nightlife group (Table 1). The median age of the nightlife group was less than that of the non-nightlife group (nightlife, 31 years [IQR 25–38]; non-nightlife, 39 years [IQR 29–51]; *P* < 0.001); men comprised three-fourth of the nightlife group (146, 74.5%). At the time of PCR testing, the severity was not different between the groups (*P* = 0.368). Majority had mild disease in both groups (nightlife, 188/196 [95.9%]; non-nightlife, 1262/1321 [95.5%]). Forty-six (3.0%) individuals had severe disease and 21 (1.4%) were asymptomatic. There was no positive test result among asymptomatic individuals. Two-thirds of the nightlife group (129, 65.8%) were workers, and the rest (67, 34.2%) were customers. In total, 82 individuals (41.8%) had worked at or visited bars and pubs: 47 (24.0%) had been to hostess clubs, 38 (19.4%) to host clubs, 11 (5.6%) to nightclubs and live music clubs, 3 (1.5%) to commercial sex businesses, 4 (2.1%) to other businesses and 11 (5.6%) to unknown establishments. The weekly number of tests in the nightlife group peaked in the week of 6–12 April. The nightlife group's proportion of positive tests was 63.8% (125/196). Within the nightlife group, the positive test proportions were not different across the exposure category (exposed, 34/52 [65.4%]; non-exposed, 91/144 [63.2%]; *P* = 0.867).

**Table 1.** Basic characteristics and SARS-CoV-2 PCR result of nightlife and non-nightlife groups before and after matching^a^

	Before matching				After matching			
	Nightlife (*n* = 196)	Non-nightlife (*n* = 1321)	Standardised mean difference	*P* value^b^	Nightlife (*n* = 196)	Non-nightlife (*n* = 196)	Standardised mean difference	*P* value^b^
Age, years	31 (25–38)	39 (29–51)	0.61	<0.001	31 (25–38)	32 (25–41)	0.0088	0.617
Sex			0.46	<0.001			0.012	1.0
Male	146 (74.5)	721 (54.6)			146 (74.5)	146 (74.5)		
Female	50 (25.5)	600 (45.4)			50 (25.5)	50 (25.5)		
Nationality			0.19	0.044			0.023	0.415
Japanese	186 (94.9)	1196 (90.5)			186 (94.9)	186 (94.9)		
Non-Japanese	10 (5.1)	125 (9.5)			10 (5.1)	10 (5.1)		
Comorbidity	22 (11.2)	228 (17.3)	0.19	0.039	22 (11.2)	20 (10.2)	0.032	1.0
Severity			0.058	0.368			0.051	0.368
Asymptomatic	0 (0.0)	21 (1.6)			0 (0.0)	0 (0.0)		
Mild	188 (95.9)	1262 (95.5)			188 (95.9)	189 (96.4)		
Severe	8 (4.1)	38 (2.9)			8 (4.1)	7 (3.6)		
Day of illness	6 (5–8)	7 (4–11)	0.43	0.200	6 (5–8)	6 (5–8)	0.097	0.136
Exposure to confirmed cases	52 (26.5)	144 (10.9)	0.37	<0.001	52 (26.5)	43 (21.9)	0.046	0.436
Overseas travel	3 (1.5)	74 (5.6)	0.33	0.013	3 (1.5)	1 (0.5)	0.083	1.0
Healthcare work	6 (3.1)	124 (9.4)	0.37	0.002	6 (3.1)	3 (1.5)	0.030	1.0
SARS-CoV-2 PCR positive	125 (63.8)	207 (15.7)		<0.001	125 (63.8)	45 (23.0)		<0.001

PCR, polymerase chain reaction; SARS-CoV-2, severe acute respiratory syndrome coronavirus 2.

a Median (interquartile range) for continuous variables, number (%) for categorical variables.

b Mann–Whitney U test for continuous variables, Fisher's exact test or Kruskal−Wallis test for categorical variables.

The nightlife and non-nightlife groups differed in age, sex, nationality, comorbidity, exposure, overseas travel and HCW (Table 1). All patients in the nightlife group were matched to similar patients in the non-nightlife group, resulting in improved covariate balance in the matched group. After matching, the proportion of positive SARS-CoV-2 PCR tests in the nightlife group was significantly higher than that in the non-nightlife group (nightlife, 63.8%; non-nightlife, 23.0%; *P* < 0.001).

In this study, exposure to nightlife businesses was significantly associated with positive SARS-CoV-2 PCR test results. This finding could reflect the nature of these businesses that many people dine and talk in closed indoor environments for several hours, as in ‘three Cs’. Most individuals in the nightlife group were young and healthy people whose clinical presentations were more likely to be mild or asymptomatic if infected with the virus. Encounters between workers and customers in this group would likely be anonymous and opportunistic. Contact tracing of the nightlife clusters has been reported to be challenging due to the nature of the business and the fear of stigma [5]. Exposure to confirmed cases among them was, therefore, less likely to be recognised or reported. In COVID-19 spread associated with nightlife businesses in Seoul, South Korea, they implemented anonymous testing to encourage and reassure nightclub visitors to have the testing [8].

Additionally, the numbers of tests and positive results in the nightlife group peaked within two weeks of the Tokyo governor's announcement about nightlife clusters on 30 March, which is consistent with the incubation period of SARS-CoV-2 infection [9]. Tokyo's strategy to focus on nightlife clusters seems reasonable, at least as one of the comprehensive strategies. Although the changes in the weekly proportion of positive tests at the NCGM and in Tokyo were not significantly different during the study period, the study results should not be overgeneralised. The patients' demography at the NCGM may have overrepresented the nightlife cohort compared to that in Tokyo as a whole. The nightlife industry has made efforts to implement infection prevention measures and establish communication with local health authorities [10]. Although strong peer pressure may have been a reason for Japan's relative success in containment so far, it should not be a hurdle for patients with suggestive symptoms to seek medical care or for their contacts to cooperate in contact tracing.

The higher proportion of positive SARS-CoV-2 PCR tests in Japan compared to other countries was criticised in the first wave of the outbreak. However, a comparison of the positive test proportions across different settings with different control strategies should be made with caution. Considering that positive results were observed in a heterogeneous manner in the NCGM cohort, it is likely that when effective cluster investigation is put in place, the overall proportion of positive tests will rise, reflecting the higher proportion in the detected clusters. Although adequate testing coverage is fundamental in the control of infectious diseases outbreaks, we still do not know what the adequate testing strategy is for COVID-19. It is unclear if certain risk groups, symptomatic individuals or everyone including asymptomatic individuals should be tested. Some countries chose open public testing that included testing asymptomatic individuals and other countries chose to test only symptomatic ones. Japan implemented a targeted testing strategy with in-depth cluster investigation and has reported fewer COVID-19 cases and deaths compared to reports of other industrialised countries [1]. To face the pandemic that does not seem to settle in the foreseeable future, we need to discuss the testing strategy to optimise our finite resources. Understanding the heterogeneity of the virus transmission in the community illustrated by this study will benefit the discussion.

This study has a few limitations. Firstly, the results of the study may not be generalisable due to the reasons discussed above. Secondly, the information was retrieved mainly from the patients' questionnaire form; therefore, the information is subject to reporting bias. Thirdly, the number of nightlife group individuals may have been underestimated.

In conclusion, exposure to nightlife businesses was significantly associated with positive SARS-CoV-2 PCR test results at the NCGM between March and April 2020. An inclusive approach to mitigate risks related to the businesses needs to be sought for future COVID-19 outbreaks.

## Data Availability

Readers can contact the corresponding author if they want access to the raw data of the study.

## Summary

Nightlife businesses were indicated as high-risk environments for severe acute respiratory syndrome-coronavirus-2 (SARS-CoV-2) transmission at the early phase of the coronavirus disease outbreak in Tokyo. We collected clinical and epidemiological information of the patients who underwent SARS-CoV-2 PCR testing at the National Center for Global Health and Medicine, Tokyo, from 9 March to 26 April 2020. A nightlife group was defined as those who had worked at or visited the businesses. The proportions of positive test results in the nightlife and non-nightlife groups were analysed using propensity score matching. We included 1517 individuals; 196 (12.9%) were categorised as the nightlife group. The proportion of positive test results in the nightlife group was significantly higher than that in the non-nightlife group after matching (nightlife, 63.8%; non-nightlife, 23.0%). Exposure to nightlife businesses was significantly associated with positive SARS-CoV-2 PCR test result. An inclusive approach is needed towards risk mitigation related to the businesses.
